# Improving resource mobilisation for global health R&D: a role for coordination platforms?

**DOI:** 10.1136/bmjgh-2018-001209

**Published:** 2019-02-27

**Authors:** Naomi Beyeler, Sara Fewer, Marcel Yotebieng, Gavin Yamey

**Affiliations:** 1 Evidence to Policy Initiative, Global Health Group, Institute for Global Health Sciences, University of California, San Francisco, California, USA; 2 Division of Epidemiology, Ohio State University, Columbus, Ohio, USA; 3 Center for Policy Impact in Global Health, Duke Global Health Institute, Duke University, Durham, North Carolina, USA

**Keywords:** health policies and all other topics, child health, control strategies, health economics, health policy

## Abstract

Achieving many of the health targets in the Sustainable Development Goals will not be possible without increased financing for global health research and development (R&D). Yet financing for neglected disease product development fell from 2009-2015, with the exception of a one-time injection of Ebola funding. An important cause of the global health R&D funding gap is lack of coordination across R&D initiatives. In particular, existing initiatives lack robust priority-setting processes and transparency about investment decisions. Low-income countries (LICs) and middle-income countries (MICs) are also often excluded from global investment initiatives and priority-setting discussions, leading to limited investment by these countries. An overarching global health R&D coordination platform is one promising response to these challenges. This analysis examines the essential functions such a platform must play, how it should be structured to maximise effectiveness and investment strategies for diversifying potential investors, with an emphasis on building LIC and MIC engagement. Our analysis suggests that a coordination platform should have four key functions: building consensus on R&D priorities; facilitating information sharing about past and future investments; building in accountability mechanisms to track R&D spending against investment targets and curating a portfolio of prioritised projects alongside mechanisms to link funders to these projects. Several design features are likely to increase the platform’s success: public ownership and management; separation of coordination and financing functions; inclusion of multiple diseases; coordination across global and national efforts; development of an international R&D ‘roadmap’ and a strategy for the financial sustainability of the platform’s secretariat.

**Summary box**

- Achieving many of the health targets in the Sustainable Development Goals will not be possible without scaling up financing for global health R&D, including product development.
- Barriers to closing the funding gap for global health R&D include a lack of coordination across global health R&D finance initiatives, lack of robust R&D priority-setting processes and insufficient transparency among R&D initiatives.
- The launch of a new global health R&D coordination platform could address these challenges by establishing consensus on R&D priorities, facilitating information sharing across investors and research institutions, building accountability mechanisms for R&D investments and curating a portfolio of prioritised R&D investment opportunities.
- To be successful, such a coordination platform must link global and national priority-setting processes across multiple diseases, develop an international ‘roadmap’ for conducting R&D and include a strategy for the financial sustainability of a publicly owned and managed platform secretariat.

## Introduction

Research and development (R&D) for new tools and technologies to combat neglected diseases (box 1) and emerging infectious diseases (EIDs) is more critical than ever. The Sustainable Development Goals (SDGs) contain a set of highly ambitious health targets to be reached by 2030 including: ‘end the epidemics of AIDS, tuberculosis, malaria and neglected tropical diseases’. Multiple analyses show that achieving these goals will not be possible with today’s health tools alone. It will require the development and delivery of new drugs, diagnostics, vaccines and other health technologies.^1–3^

**Box 1 Neglected diseases**

In this policy paper, we use the term neglected diseases to refer to the list of 35 diseases or conditions defined by the independent research group Policy Cures Research (PCR, http://www.policycuresresearch.org) as neglected. This list, which is at https://gfinder.policycuresresearch.org/staticContent/pdf/ND_matrix.pdf, is used in PCR’s annual G-FINDER reports, which estimate annual funding for neglected disease product development. PCR uses three key principles in defining a disease as neglected: it disproportionately affects LICs and MICs worldwide, there is a need for new products and there is market failure. The list includes HIV, tuberculosis, malaria, pneumonia, diarrhoea, neglected tropical diseases and reproductive health needs of LICs and MICs.

There is a paradox at the heart of the SDG agenda.^4^ The health targets have become more ambitious and yet, with the exception of a one-off injection of funding for Ebola following the 2014 outbreak, financing for neglected disease product development fell steadily from 2009-2015.^5^

A recent study by Young and colleagues examined the current pipeline of candidate products for neglected diseases,^6^ using a new financial modelling tool, the Portfolio-to-Impact (P2I) tool,^7^ to estimate the costs to move these candidates through the pipeline, the likely resulting launches and the needed products that would still be ‘missing’ at the end. The study found that, based on what is currently in the pipeline, launches of highly efficacious vaccines for HIV, TB, malaria or hepatitis C would be unlikely, as would launches of a complex new chemical entity (NCE) for TB and NCEs for several neglected tropical diseases. It estimated that the annual funding gap for neglected disease product development over the next 5 years is at least $1.5–1.8 billion.

One major barrier to closing the funding gap for neglected diseases and EIDs is a lack of coordination across global health R&D finance initiatives. In 2010, the WHO Expert Working Group on Research and Development Financing found that the field of global health R&D is highly fragmented, with some informal governing arrangements but no global coordination mechanism.^8^ The 2012 WHO Consultative Expert Working Group (CEWG) on Research and Development: Coordination and Financing also highlighted the importance of coordination in optimising R&D financing and resource allocation.^9^ Global health R&D experts have continued to call for improved global coordination for R&D financing, but this has not been fully realised.^10–13^ In fact, while the Ebola outbreak represented one of the more substantial increases in R&D funding in recent history, the lack of effective coordination and governance is cited as one reason for the delayed and ineffective response to that outbreak.^14^

In this paper, we identify the main challenges posed by a lack of coordination for global health R&D. We then outline a set of policy options for the design of an effective coordination platform for R&D investments focusing on the essential functions such a platform must play and how it should be structured to maximise its effectiveness. We explore strategies to diversify the range of funders who might invest in an R&D platform, with an emphasis on building engagement of LICs and MICs. Our analytic approach is summarised in box 2. We hope this policy paper provides a roadmap for effectively leveraging coordination platforms to increase the value of current investments and close the funding gap for global health R&D.

**Box 2 Our analytic approach**

We reviewed academic and grey literature, including reports on nascent R&D coordination initiatives, to identify trends, mechanisms and challenges in global health R&D financing. Our search included keywords related to the categories of global health research and development, global health finance and R&D finance, coordination and governance. We limited our literature search to articles published after 2012 given the rapid growth of new R&D platforms in the subsequent years. Our aim was to identify (1) which functions, structures and strategies are needed to mobilise, prioritise and coordinate health R&D finance at the global and regional level and (2) which strategies could help to incentivise participation of LICs and MICs in resource mobilisation and coordination activities. We also spoke with R&D experts from institutions and organisations at the global level and in MICs, that fund, conduct or coordinate global health R&D.

The initial findings from this approach were discussed during two policy workshops. The first was a 2-day workshop, hosted by the Duke University Center for Policy Impact in Global Health, with representatives from government agencies, philanthropic donor agencies, global health multilateral agencies, research institutions, academia and think tanks. The second was a policy salon on accelerating the development of medical products, hosted by the National Academies of Sciences, Engineering, and Medicine (NASEM; two of this paper’s authors, Gavin Yamey and Marcel Yotebieng, were panellists at this event). The salon was a dissemination event stemming from the Academies' recent report on Global Health and the Future Role of the United States.^29^ Both sessions included representatives from national governments and R&D institutions in several LICs and MICs, and one author of this paper (MY) is from an LIC. The discussions at these engagements informed this policy options paper. Our analysis mostly focuses on product development for neglected diseases and emerging infectious diseases, though the key findings also apply to a broader range of research, including population, policy and implementation research.

## Challenges caused by a lack of coordination

Our analysis identified three main challenges that stem from the lack of coordination of R&D investments and activities, which limit the scale and effectiveness of these investments.

### Lack of a clear global health R&D priority setting process

In recent years, a number of new initiatives have been launched to spur greater funding for global health R&D (table 1). These initiatives leverage investments across the public, private and philanthropic sectors to address financing gaps at various parts of the development pipeline. However, each initiative addresses a specific piece of the R&D pipeline, without an overarching framework that identifies which diseases, products and phases of development need the most support across the range of all neglected diseases and EIDs.

**Table 1:** Key funding mechanisms for global health R&D, launched 2013–2016

Funding mechanism	Launch year	Partners	Description	Funding mobilised (by 2018)
Global Health Investment Fund (GHIF)	2013	Bill & Melinda Gates Foundation, J.P. Morgan	Mobilises capital from high-net worth individuals and institutions to fund late-stage innovations for neglected diseases, seeking social impact and a return on investment	US$108 million
Global Health Innovative Technology Fund (GHIT Fund)	2013	Japanese government, Japanese pharmaceutical companies, Bill & Melinda Gates Foundation, Wellcome Trust, United Nations Development Programme	Invests in the discovery, preclinical, and other development phases of neglected disease projects, including HIV/AIDS, malaria, TB and NTDs	US$345 million committed (US$145 million in funding from 2013 to 2017 and US$200 million secured for 2018–2020)
Combating Antibiotic Resistant Bacteria Biopharmaceutical Accelerator (CARB-X)	2016	Wellcome Trust, US Department of Health and Human Services Biomedical Advanced Research and Development Authority, US National Institute of Allergy and Infectious Disease, UK Department of Health and Social Care, Bill & Melinda Gates Foundation, Boston University	Provides grants, scientific and business support to advance the early stages of innovative antibiotics and other therapeutics, vaccines, rapid diagnostics and devices to address drug-resistant bacterial infections	$500 million from 2016 to 2021
Global Antibiotic Research and Development Partnership (GARDP)	2016	WHO, Drugs for Neglected Diseases initiative	Identifies gaps in the antibiotic pipeline and partners with research institutions and pharmaceutical companies to advance product development, particularly of new therapeutics	US$65 million pledged
Coalition for Epidemic Preparedness Innovations (CEPI)	2016	Bill & Melinda Gates Foundation, Wellcome Trust, World Economic Forum, European Commission and the Governments of Australia, Belgium, Canada, Germany, India, Japan, and Norway	Focuses on pre-outbreak vaccine development for priority diseases from the WHO R&D Blueprint for Action to Prevent Epidemics	US$630 million raised of US$1 billion goal

NTD, neglected tropical disease.

The lack of clear global priorities and strategies contributes to inefficient investments and adversely impacts advocacy and resource mobilisation efforts. Without clear guidance from global institutions, potential funders do not have access to critical information that would enable them to choose between investment options. Disease-specific priority setting activities can contribute to competition for scarce resources; unlike more comprehensive multi-disease priority setting processes, disease-specific efforts can overwhelm R&D investors with conflicting information about global investment needs.

Recent priority setting exercises also raised questions about the robustness of current processes. Two recent exercises led to the exclusion of critical diseases, creating confusion and raising questions about the credibility of international guiding bodies: the exclusion of TB from the WHO’s global priority list of antibiotic-resistant bacteria^16^ and the exclusion of influenza from the WHO R&D Blueprint for Action to Prevent Epidemics.^17^

### Insufficient transparency and information sharing among existing R&D initiatives

There is little information sharing that allows investors or research institutions to:

- identify who is funding which activities,
- assess and share the findings of completed and ongoing research, including successes and failures,
- assess and promote efforts that appear promising for future research or
- link funders to promising projects to speed development.

Such information sharing is crucial to align funding with priority projects and to avoid gaps and duplication in funding.^18^

Several new coordination platforms have recently launched that aim to address these types of information gaps (table 2). While promising, these coordination initiatives operate on small budgets with few staff, and there is still no overarching, inclusive platform that systematically collects all the required information to properly coordinate global health R&D investments and activities.

**Table 2:** Newly launched global coordination mechanisms for global health R&D

Coordination mechanism	Launch year	Partners	Description
Coalition for African Research and Innovation	2017	African scientific thought leaders, international funders and global industry leaders	Sets priorities for and spurs innovation to meet regional R&D needs
Global Research Collaboration for Infectious Disease Preparedness	2013	27 of the world’s major research funders, the Coalition for Epidemic Preparedness Innovations and the WHO	Brings together funding bodies to facilitate an effective research response to disease outbreaks with pandemic potential
WHO Global Observatory on Health R&D	2017	Funding partners include the European Commission, France, Germany, Switzerland, USA	Identifies global health R&D priorities by monitoring and analysing health R&D needs, collecting data and supporting coordination
Global Antimicrobial Resistance Research and Development Hub	2018	18 members including Germany, Russia, China, USA, France, Bill & Melinda Gates Foundation, Wellcome Trust, European Commission	Mobilises funding for R&D for new treatments and diagnostics for resistant pathogens

R&D, research and development.

### Lack of diversified funding sources, which limits sustainable finance for global health R&D

**Figure 1:**
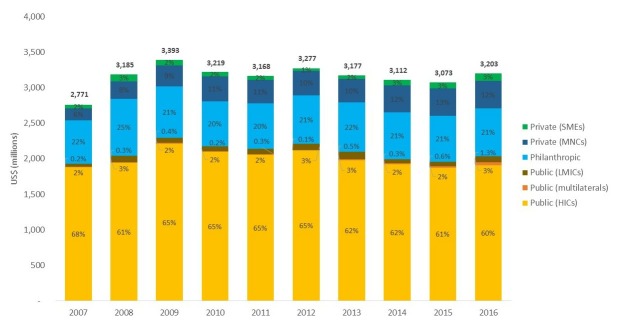
Funding for neglected disease product development by sector, 2007–2016. Figure from Ref. 5. HICs, high-income countries; LMICs, low-income and middle-income countries; MNCs, multinational pharmaceutical companies; SMEs, small pharmaceutical and biotechnology firms.

Funding is driven by a small number of high-income country (HIC) governments and philanthropists, particularly the US government, which contributed 47% of all funding for neglected disease product development in 2016. In contrast, investments by LICs and MICs made up just 3% of R&D funding in the same year (figure 1).^5^ Expanding and diversifying public and private investments is required to reduce overdependence on a small number of funders and support the long-term investments needed to drive sustainable innovation and meet the needs of highest-burden countries. The global community is looking to LICs and MICs to step up their R&D contributions (box 3). However, calls for increased funding from LICs and MICs have not been met,^9^ largely because (1) these countries have often been excluded from global investment strategy and priority-setting discussions, (2) current funding initiatives are perceived as models invented by and for HICs and (3) such initiatives do not incentivise LIC and MIC participation.

Box 3 **Making the case for LICs and MICs to invest in R&D for neglected diseases**

Several MICs, such as Brazil, China and India, have started to invest in R&D, which could have substantial economic benefits within these countries.^30 31^ India was the fourth and Brazil was the ninth largest funder of neglected disease product development in 2016.^5^ There are benefits to these countries in continuing and increasing their commitments to R&D, and other LICs and MICs may experience similar success by stepping up their R&D investments. However, stakeholders that participated in our interviews and workshops noted a need to better articulate the investment case to high-level policymakers in LICs and MICs to create enabling environments for national R&D investments.

Research centres can serve as hubs for innovation, offer employment, attract investment and produce evidence to help policymakers address public health priorities. These potential returns on investment must be better articulated, including the near-term benefits for ministries of finance and politicians and the long-term public health and security value of locally relevant R&D. As LICs and MICs invest more in R&D, these public funds could be used to mobilise private finance, such as by reducing the financial risk facing emerging R&D industries and attracting new pharmaceutical and biotech partners.

With universal health coverage (UHC) now included as a target in the SDGs, governments of LICs and MICs have a window of opportunity to commit a percentage of domestic health budgets to R&D as a strategy to help reach UHC. Indeed, the 2013 World Health Report, Research for Universal Health Coverage, argued that UHC cannot be achieved without R&D and that ‘all nations should be producers of research as well as consumers’.^32^

Public sector HIC investments dominate R&D funding, but are unlikely to expand given recent trends in development assistance for health, which has largely stagnated since the 2008 global financial crisis.^19^ Along with expanded investment from LICs and MICs, greater private and philanthropic investment is critical to closing R&D financing gaps.

## Key coordination functions

Current platforms play only a subset of the coordination functions considered essential to maximise investments. For example, the WHO Global Observatory on Health R&D and the G-FINDER survey track R&D finance globally, but do not include mechanisms to (1) hold donors or other investors accountable for meeting stated commitments to R&D finance or (2) incentivise investors to ratchet up investments in line with global need. Likewise, product development partnerships establish investment priorities for specific diseases, but the priorities across the whole portfolio of neglected diseases and EIDs remain unclear.

There is an important role for a new, overarching global health R&D funding and coordination platform. Our analysis suggests that such a platform must meet the following four coordination functions:

### Set R&D priorities

**Figure 2:**
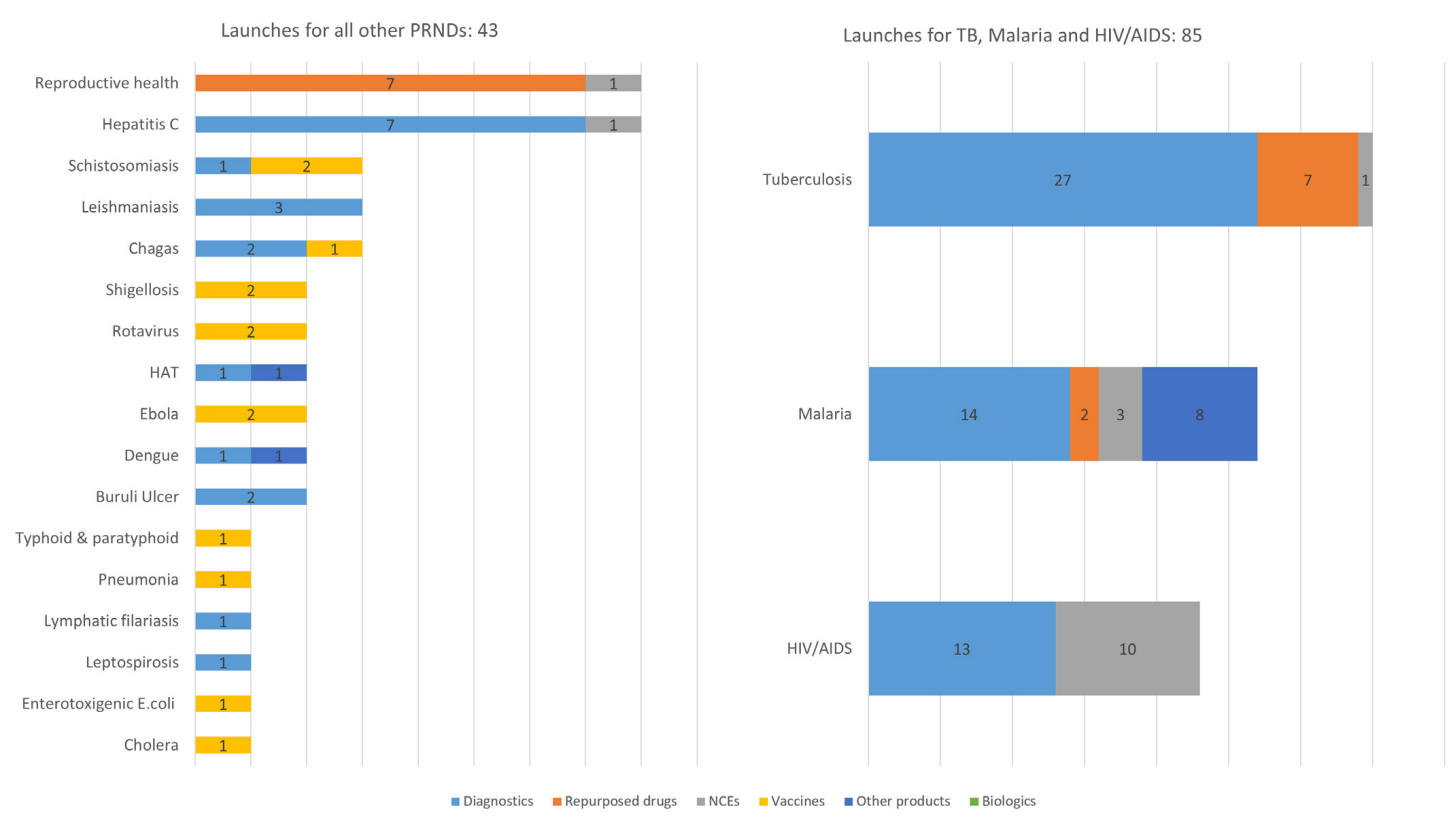
Breakdown of estimated product launches by 2030, by disease and product type. Other products comprises vector control products (eg, insecticide-treated bed nets). Figure from Ref. 7. HAT, human African trypanosomiasis; NCEs, new chemical entities; PRNDs, poverty-related and neglected diseases.

Processes for setting R&D priorities must be more transparent and engage a broader group of stakeholders. These processes should emphasise engaging LICs and MICs to set priorities that respond to country health needs, the barriers and opportunities for innovation and the uptake of such innovation at country level. Global priority-setting activities should be accompanied by regional priority-setting processes. These activities must consider multiple diseases together in order to prevent fragmentation of limited resources and to maximise investments with cross-disease benefits. The P2I tool can be used to assess the current pipeline of product candidates for neglected diseases and EIDs and to estimate the costs and likely launches associated with moving candidates through this pipeline.^7^ The tool can show where the pipeline is strongest and weakest. For example, Young and colleagues used this tool to estimate that there would be around 128 product launches resulting from the current pipeline of candidates, but these would not be ‘balanced’ across the portfolio of 35 neglected diseases (figure 2).^6^

### Facilitate information sharing

Coordination platforms must incentivise funders, product developers, industry and researchers to share information about projects in the pipeline, past successes and failures and anticipated funding portfolios. This will minimise the likelihood of gaps and duplication in funding and support funders in making efficient investment decisions. Information-sharing mechanisms should include real-time updating processes to ensure transparency and ongoing alignment between global goals and global finance. These mechanisms should also support opportunities for North-South and South-South learning across investors, public sector leaders and R&D institutions. For example, the Global Research Collaboration for Infectious Disease Preparedness (GloPID-R) is a network of global research institutions and funders designed to improve pandemic research through data sharing during disease outbreaks and information sharing across funders about relevant ongoing research.^20^ Innovations in coordinating pandemic research efforts could be expanded to address a broader range of diseases.

### Build in accountability mechanisms

Coordination platforms must put in place systems that encourage sustained investments that are well aligned with established global and regional R&D priorities. Such mechanisms should include:

- regular tracking and reporting of R&D spending to assess progress towards priority investment targets,
- established funding norms and guidelines that platform members agree to and
- accountability systems that track funder alignment with established goals and priorities.

R&D investments are poorly aligned with the priority needs in LICs and MICs.^21^ A recent study of randomised controlled trials globally found that in sub-Saharan Africa and South Asia, there was substantial mismatch between the number of research studies conducted by disease and the burden of disease in each country.^22^ A coordination platform should establish systems through which donors will have greater accountability to meet needs prioritised at the national and global level, minimising such mismatch moving forward. At the same time, accountability mechanisms must be developed alongside investment incentives such that private donors and investors see adequate value in participation.

### Facilitate funder and researcher partnerships

Coordination platforms should play a central role in creating mechanisms to link funders to a portfolio of prioritised and promising projects. This role is particularly important for smaller funders and countries that may not have the capacity to research and identify promising investments but could be incentivised to invest in a well-curated portfolio with promising returns. A brokering function such as this could also facilitate new donors, including private and individual investors, to enter the global health R&D space. For example, the Every Woman Every Child Innovation Marketplace, hosted by Grand Challenges Canada, provides investors with a vetted and curated set of investment opportunities, thereby minimising transaction costs of finding and evaluating a high volume of high value projects.^23^

## Key structural considerations for coordination mechanism

To be effective in achieving the above key functions, a global R&D coordination platform must be structured such that it has high legitimacy and trust from donors, R&D institutions and LICs/MICs. The following six recommendations outline features of platform design and structure that are likely to foster long-term success.

### Develop broad-based and public ownership and management

The success of a coordination platform will depend on the willingness of a diverse coalition of stakeholders to participate in and follow the norms and guidance of such a platform. To build the engagement of LICs and MICs, coordination platforms must be hosted in an organisation that has political legitimacy and that LICs and MICs trust. Coordination platforms must also be led by, owned by and accountable to the public sector. Public sector funding makes up the large majority of R&D funding; there is some concern among global health R&D experts about the outsized role of private philanthropy in driving priority and strategy conversations without sufficient public input.

### Separate coordination and financing functions

It is unlikely that a single platform can effectively coordinate R&D strategy and implementation as well as mobilise and manage investments. Recent pooled funds have had variable success given the preference of donors to maintain greater control over their investments. If separate pooled funding initiatives continue, coordination platforms should ideally incentivise donors to align investments to global priorities, while not constraining investments. For coordination platforms to maintain political and technical legitimacy, these platforms should not play a leading role in advocacy for R&D resource mobilisation.

### Create multidisease platforms

Current R&D platforms are disease-specific, which enables deep technical expertise and targeted advocacy campaigns tailored to the needs of that disease.^21^ However, developing multiple disease-specific platforms carries risks for overall health R&D as this approach (1) creates a fragmented funding environment where platforms compete for limited funds, (2) misses opportunities for basic science investments with benefits across multiple diseases and (3) threatens the success of and adherence to global priorities and strategies. A global coordination platform should serve as an organising body to establish priorities *across* disease and issue areas. There are several new platforms such as GloPID-R and CEPI that establish priorities across a set of diseases. However, these platforms still focus on a relatively narrow group of diseases and do not facilitate priority-setting across the global health R&D space. A broader coordination platform should facilitate coordination across both disease***-***specific investment and existing multidisease platforms as well as collaborate with the diversity of disease-specific advocacy communities, to ensure alignment of funds with global priorities.

### Pair global and national efforts

A global coordination platform must engage LICs and MICs in R&D priority-setting and align with the health priorities of these countries. Yamey and colleagues argued, in an editorial reporting on the NASEM policy salon, that donors must ‘work more closely with experts within low and middle income countries to tackle the documented mismatch between global and national research priorities’.^24^ Greater engagement of LICs and MICs in a shared prioritisation process could help to make R&D more needs driven. The international community can enable this by strengthening country scientific capacity, such as by: (1) engaging researchers in LICs and MICs to develop and produce cost-sensitive innovations that target national priorities; (2) funding and strengthening the capacity of local investigators to lead research and (3) building strong clinical trial sites for multiple diseases. The international community can also support countries in strengthening their civil registration, vital statistics and surveillance systems (CRVS), which are critical for identifying global R&D needs, as shown by the important role Brazil’s strong CRVS played in the timely identification of the 2015 Zika outbreak. There is a crucial role for regional institutions, such as the Africa Centres for Disease Control and Prevention, in facilitating this coordination and capacity building.^24^

### Develop an international ‘roadmap’ for conducting R&D

Such a roadmap would be ‘an analysis of the current capacities of all countries, the steps each country should take to increase its capacity, and the costs of such improvement’.^24^ It would cover all stages of R&D on neglected diseases and EIDs, from basic science to clinical research to implementation research. Along with the roadmap, there should be a set of indicators of progress. Yamey and colleagues argue that such a roadmap could help donors to better target their R&D capacity-building efforts in LICs and MICs and would be valuable to guide the efforts of the new funding initiatives shown in table 1.^24^

### Develop a strategy for the sustainability of the platform’s secretariat

Adequate funding for the platform’s operation is needed. Existing platforms such as the WHO Observatory are hindered by a lack of support for the platform’s core coordination functions (which is distinct from direct investments in R&D). Without a dedicated and long-term source of finance, existing and new coordination platforms will be limited in their ability to provide ongoing global and regional agenda-setting, up-to-date curation and sharing of information about the pipeline and meaningful facilitation of partnerships and accountability systems. As noted in the aftermath of the Ebola outbreaks, there is a cycle of ‘panic and neglect’—swelling funding and interest during on outbreak that is not maintained between emergency events.^25^ An effective response will require sustained investment and research at the national and global level, supported by a coordination platform that can maintain these efforts between emergencies.

## Conclusion

To achieve many of the health targets in the SDGs by 2030, the global health community must urgently mobilise new financing and better leverage existing investments for global health R&D. Recently established initiatives to support global health R&D projects must be complemented with improved coordination. Recent analysis of the global health financing landscape shows that the growth of voluntary contributions in multilateral organisations has enabled donors to have greater control over how funds are allocated and used, while also expanding discretionary, shorter-term and vertical initiatives.^26^ Our analysis finds that a global coordination platform could ensure such initiatives are aligned both within and across disease focus areas, by helping to clarify investment priorities across neglected diseases and EIDs, increase transparency around investments and expand and diversify stakeholders to improve alignment of investment and need, particularly by engaging LICs and MICs. The lessons learnt regarding coordination for neglected disease and EIDs could also be applied to other global health challenges. Lack of coordination is one of the main critiques of the current development assistance system, and indeed coordination has been proposed as a solution to increase aid effectiveness across multiple health issues.^27 28^ Our analysis provides an important overview of the functional and structural considerations that could be applied to the development of such coordination efforts. This analysis also identifies areas where additional research is needed to inform the effective creation and management of a coordination platform, including a more in-depth analysis of specific and effective accountability mechanisms, strategies for engaging and overseeing private sector investors and approaches for building and supporting LIC and MIC leadership. Nonetheless, it is clear that the global community has an important opportunity to design a platform that better links national needs to the global agenda and engages LICs and MICs in global health R&D decision-making and funding.
